# Detection of Photosynthetic Performance of *Stipa bungeana* Seedlings under Climatic Change using Chlorophyll Fluorescence Imaging

**DOI:** 10.3389/fpls.2015.01254

**Published:** 2016-01-12

**Authors:** Xiliang Song, Guangsheng Zhou, Zhenzhu Xu, Xiaomin Lv, Yuhui Wang

**Affiliations:** ^1^State Key Laboratory of Vegetation and Environmental Change, Institute of Botany, Chinese Academy of ScienceBeijing, China; ^2^University of Chinese Academy of SciencesBeijing, China; ^3^Chinese Academy of Meteorological Sciences, China Meteorological AdministrationBeijing, China

**Keywords:** *Stipa bungeana*, chlorophyll fluorescence imaging, photosynthetic efficiency, energy partitioning, high temperature, precipitation change

## Abstract

In this study, the impact of future climate change on photosynthetic efficiency as well as energy partitioning in the *Stipa bungeana* was investigated by using chlorophyll fluorescence imaging (CFI) technique. Two thermal regimes (room temperature, T_0_: 23.0/17.0°*C*; High temperature, T_6_: 29.0/23.0°*C*) and three water conditions (Control, W_0_; Water deficit, W_−30_; excess precipitation, W_+30_) were set up in artificial control chambers. The results showed that excess precipitation had no significant effect on chlorophyll fluorescence parameters, while water deficit decreased the maximal quantum yield of photosystem II (PSII) photochemistry for the dark-adapted state (*F*_v_/*F*_m_) by 16.7%, with no large change in maximal quantum yield of PSII photochemistry for the light-adapted state (*F*_V_′/*F*_M_′) and coefficient of the photochemical quenching (*q*_*P*_) at T_0_ condition. Under T_6_ condition, high temperature offset the negative effect of water deficit on *F*_v_/*F*_m_ and enhanced the positive effect of excess precipitation on *F*_v_/*F*_m_, *F*_v_′/*F*_m_′, and *q*_*P*_, the values of which all increased. This indicates that the temperature higher by 6°*C* will be beneficial to the photosynthetic performance of *S. bungeana*. Spatial changes of photosynthetic performance were monitored in three areas of interest (AOIs) located on the bottom, middle and upper position of leaf. Chlorophyll fluorescence images (*F*_v_/*F*_m_, actual quantum yield of PSII photochemistry for the light-adapted state (Φ_PSII_), quantum yield of non-regulated energy dissipation for the light-adapted state (Φ_NO_) at T_0_ condition, and Φ_PSII_ at T_6_ condition) showed a large spatial variation, with greater value of Φ_NO_ and lower values of *F*_v_/*F*_m_ and Φ_PSII_ in the upper position of leaves. Moreover, there was a closer relationship between Φ_PSII_ and Φ_NO_, suggesting that the energy dissipation by non-regulated quenching mechanisms played a dominant role in the yield of PSII photochemistry. It was also found that, among all measured fluorescence parameters, the *F*_v_/*F*_m_ ratio was most sensitive to precipitation change at T_0_, while Φ_PSII_ was the most sensitive indicator at T_6_.

## Introduction

High temperature and water stress as abiotic stress factors will limit plant growth and reduce crop productivity (Boyer, 1982; Wahid et al., 2007), and they always occur simultaneously in that high temperature increases both evaporation and potential evapotranspiration and exacerbates the negative influence of water deficit (Machado and Paulsen, 2001; Osório et al., 2011). Models of global climate change have predicted that the globally averaged surface temperature will be 1.5–4.0°*C* higher till 2100 and the extreme precipitation events will occur more frequently than before (IPCC, 2013). According to the study by Xu et al. (2009), temperature and precipitation change determine the physiological response of perennial grass to new environmental conditions to a large extent. Among all plant physiological functions, photosynthesis plays a pivotal role in plant carbon uptake, plant growth and biomass accumulation. It is commonly considered that stomatal limitation which influences the substomatal CO_2_ concentration is the main reason for the reduction of photosynthesis under moderate water deficit (Cornic, 2000). The limitation on CO_2_ assimilation may damage the balance between photochemical activity in photosystem II (PSII) and electron requirement for photosynthesis, resulting in the photodamage of PSII centers. Although plant photosynthetic apparatus appears to be highly resistant to water deficit (Giardi et al., 1996; Petsas and Grammatikopoulos, 2009; Zivcak et al., 2014), temperature rising can change the response of photosynthesis to water stress (Chaves et al., 2002). Among all plant physiological activities, photosynthesis has been proved to be most sensitive to high temperature and can be inhibited entirely by heat stress before other plant physiological symptoms occur (Berry and Bjorkman, 1980). High temperature damages several photosynthetic functions, such as Calvin cycle, photosystem I (PSI) and PSII. Many studies have reported that the cooperative effect of water stress and high temperature is more drastic than their single effect (Albert et al., 2011; Thomey et al., 2011; Bauweraerts et al., 2013). When water and heat stress occur simultaneously, water stress may impose a certain effect on the photosynthesis together with temperature through oxidative damage (Chaves et al., 2002). On this basis, the inhibitory effect and damage on photosynthesis can be studied when the two stresses coexist, even at a low light intensity.

For the quantitative detection of the changes in the photosynthetic apparatus and photosynthetic activity under various environmental stresses, chlorophyll fluorescence measurement has been demonstrated to be a fast, non-destructive, sensitive and reliable method (Berry and Bjorkman, 1980; Havaux, 1992; Martínez-Carrasco et al., 2002; Mielke et al., 2003; Xu et al., 2004; Xu and Zhou, 2006; Swoczyna et al., 2010; Tuba et al., 2010; Ogaya et al., 2011; Brestic et al., 2014; Kalaji et al., 2014; Lazár, 2015). However, the conventional chlorophyll fluorescence measurement approach is based on point measurements and cannot exhibit the physiological status of a whole plant (Lichtenthaler and Miehé, 1997; Ehlert and Hincha, 2008). Furthermore, habitual heterogeneity of photosynthetic activity over the leaf surface makes this approach highly error-prone (Ehlert and Hincha, 2008). To overcome these problems, a more advanced technique, chlorophyll fluorescence imaging (CFI), was developed to take a powerful role in identifying spatial heterogeneity of leaf photosynthetic performance (Omasa et al., 1987; Baker and Rosenqvist, 2004; Ivanov and Bernards, 2015). This provides new possibilities to understand the regulation mechanism of photosynthesis, and to assess the properties of the photosynthetic apparatus and the extent to which the plants are affected by different stresses (Gorbe and Calatayud, 2012; Shaw et al., 2014; Humplík et al., 2015; Ivanov and Bernards, 2015). One of the first works on experiments with CFI were carried out by Omasa et al. (1987). In their work, the analysis of CFI was proved to be a useful method in early warning diagnosis, functional analysis of disorders during environment stress and plant's ability to recover. CFI can also be used to study plant response to dynamic climate control as image information is the most intuitive, easily comprehensible, and provides useful information on plant status (Omasa, 1990; Calatayud et al., 2006; Gorbe and Calatayud, 2012). Because CFI detects fluorescence signal pixel-by-pixel, it also provides huge amount of data which can be used for a sophisticated statistical treatments which can lead to an early detection of plant stress (Lazár et al., 2006).

As a key vegetation type dominating the typical steppe in Loess Plateau, *Stipa bungeana* is a useful plant species which can control water loss and soil erosion and improve the ecological environment effectively for its developed root system. It is also a type of appetizing forage with high nutritive value for livestocks. Hence, the research concerning the photosynthetic physiological responses of *S. bungeana* to the major stresses becomes increasingly important in the context of the predicted future climatic changes. In the present study, it was confirmed that CFI is a useful and convenient method for detecting the physiological mechanism of response to higher temperature and precipitation change. Moreover, the spatial variations of chlorophyll a (Chl a) fluorescence parameters of *S. bungeana* under different environmental stresses were analyzed. This work aims to evaluate the impact of high temperature and precipitation change on the photosynthetic performance and the utilization of excess excitation energy in photosynthetic apparatus of *S. bungeana*. Specifically, the following questions were addressed: (1) Are there any negative or positive impact of high temperature and precipitation change on the photosynthetic apparatus of *S. bungeana*? (2) What are the mechanisms of PSII photoprotection for *S. bungeana*? (3) Which is the most sensitive fluorescence parameter in predicting the impact of future climate change on *S. bungeana*?

## Materials

### Plant material and growth conditions

To understand the effects of high temperature and precipitation change on *S. bungeana*'s photosynthesis characteristics, the changes of water and heat conditions were controlled for the seedlings germinated from seeds. The experiment was carried out at the Institute of Botany, Chinese Academy of Sciences. The seeds of *S. bungeana* were obtained from the grassland in Dongsheng (39°82′N, 110°00′E), Inner Mongolia. They were sterilized by soaking in 0.7% potassium permanganate solution for 8 min and rinsed. Then, these seeds were sown in plastic pots wrapped with plastic film. Each plastic pot was filled with 4.08 kg of dry soil and planted with four plants. In the chestnut soil, the organic carbon content was 12.3 g·kg^−1^ and the total nitrogen content was 1.45 g·kg^−1^. Polyethylene pots were used as the experimental containers, which were lined with plastic bags to prevent water leakage.

Different temperature and precipitation treatments were set according to the monthly average temperature and precipitation during *S. bungeana* 's blooming stage in the past 30 years (1978-2007). Considering the diurnal temperature variations, two temperature treatments 23.0/17.0°*C* (T_0_) and 29.0/23.0°*C* (T_6_) were selected for experiment. Three precipitation regimes were set: average monthly precipitation over 30 years (W_0_: 82.3 mm); the average increased by 30% (W_+30_); the average decreased by 30% (W_−30_). All the plants were grown in a naturally illuminated glasshouse (the CO_2_ concentration was maintained at 390 ppm with a photosynthetic photon flux density of 1000 μmol photons m^−2^·s^−1^) and the timing used for day/night regime was 16 h light/8 h dark.

### Imaging of chlorophyll fluorescence measurement

In order to investigate the spatial heterogeneity of Chl fluorescence parameters, Chl fluorescence imaging of leaves was performed by using an imaging-PAM fluorometer (Walz, Effeltrich, Germany). Chl a fluorescence parameter was measured in the healthy and fully expanded leaves of three plants from each treatment. To evaluate spatial heterogeneity, three areas of interest (AOI, AOI type: Rectangle) in the same leaf were selected, the first one in the bottom position of the leaf (AOI1), the second one in the middle position (AOI2) and the third one in the upper position (AOI3). Three replicates of each plant were used for AOI determination. All plants were placed in dark for 10 min before measurement. Images of maximum fluorescence in the dark-adapted state, *F*_m_, was determined by applying a blue saturation pulse. The saturation pulse intensity was 8000 μmol photons m^−2^·s^−1^ for 0.8 s. Minimum Chl fluorescence yield *F*_0_ was determined using low frequency light pulses (0.5 μmol photons m^−2^·s^−1^). Then the images of maximal quantum yield of PSII photochemistry for the dark-adapted state *F*_v_/*F*_m_ were captured, and *F*_v_/*F*_m_ ratio were obtained as $\frac{F_{\text{m}}-F_{0}}{F_{\text{m}}}$. To determine the maximum fluorescence yield in the light-adapted state (*F*_m_′) and Chl fluorescence during actinic illumination (*F*_s_), actinic illumination (336 μmol photons m^−2^·s^−1^) was switched on and saturating pulses were applied at 20 s intervals for 5 min. All the fluorescence levels for the light-adapted state of the sample were determined at the end of 5 min. The maximal quantum yield of PSII photochemistry for the light-adapted state was estimated by the *F*_v_′/*F*_m_′ and was calculated by measuring the above same parameters (*F*_0_′ and *F*_m_′) on light-adapted leaves. In light-adapted state, the *F*_0_′ level was estimated using the approximation of Oxborough and Baker (1997): $F_{0}^{{}^{\prime }}=\frac{F_{0}}{\left(F_{\text{v}}/F_{\text{m}})\text{+}(F_{0}/F_{\text{m}}^{{}^{\prime }}\right)}$. The actual quantum yield of PSII photochemistry for the light-adapted state (Φ_PSII_) could be calculated by the formula: $\Phi_{\text{PSII}}=\frac{F_{\text{m}}^{{}^{\prime }}-F_{\text{s}}}{F_{\text{m}}^{{}^{\prime }}}=\frac{\Delta F}{F_{\text{m}}^{{}^{\prime }}}$ (Genty et al., 1989). The coefficient of the photochemical quenching (*q*_*P*_), which was used for the estimation of the fraction of open PSII centers, was calculated as: ${\text{q}}_{\text{P}}=\text{1}-\left(\frac{F_{\text{s}}-F_{0}^{{}^{\prime }}}{F_{\text{m}}^{{}^{\prime }}-F_{0}^{{}^{\prime }}}\right)$ (Bilger and Schreiber, 1987). The quantum yields of PSII photochemical energy dissipation (Φ_*PSII*_), non-regulated (Φ_NO_), and regulated (Φ_NPQ_) thermal energy dissipation for the light-adapted state could be used to reflect the utilization of photons which are absorbed by the PSII antennae (Lazár, 2015). It has been proved that Φ_PSII_ + Φ_NPQ_ + Φ_NO_ = 1 (Hendrickson et al., 2004; Kramer et al., 2004; Lazár, 2015). Φ_NO_in PSII was calculated by the equation $\Phi_{\text{NO}}=\frac{1}{\left[\left(\text{NPQ}+1+q_{\text{L}}\right)\left(\frac{F_{\text{m}}}{F_{0}}-1\right)\right]}$ and ΦNPQ was calculated by $\Phi_{\text{NPQ}}=1-\Phi_{\text{PSII}}-\frac{1}{\left[\left(\text{NPQ}+1+q_{\text{L}}\right)\left(\frac{F_{\text{m}}}{F_{0}}-1\right)\right]}$, separately. At last, it should be noted that all the chlorophyll fluorescence parameters were calculated by the Imaging Win v2.32 software.

### Statistical analysis

All statistical analysis was performed using SPSS 18.0 (SPSS, Chicago, Illinois, USA). The mean with standard deviation (±SD) of each treatment was shown. The parameters were analyzed by One-/Two-way analysis of variance (ANOVA) followed by Duncan's multiple range test (Duncan, 1955). The graphing were performed using Origin 9.0 software (Origin Lab, USA).

## Results and discussion

In this study, fluorescence imaging technique was used to provide real-time information of photosynthetic performance of *S. bungeana* under different heat and water conditions. The change of images revealed the spatial variation of photosynthetic efficiency in the leaves of *S. bungeana* under different climate environments.

### Chlorophyll fluorescence parameters in temperature warming and precipitation change leaves

Different chlorophyll fluorescence parameters were measured for the leaves of *S. bungeana* to determine the impact of high temperature and precipitation change on the photosynthesis. Maximal quantum yield of PSII photochemistry for the dark-adapted state (*F*_v_/*F*_m_) has been widely used as an indicator of environmental stress. It can reveal the potential electron transport of maximal PSII quantum yield in the dark-adapted state. The imaging of *q*_*P*_ and *F*_v_′/*F*_m_′ facilitates the evaluation of their variations (Oxborough and Baker, 1997). The fraction of the open PSII can be quantified by the parameter *q*_*P*_ (Lazár, 2015). The light-induced non-photochemical quenching is a process that regulates energy conversion in PSII to protect plants from photoinhibition. It represents the plant's ability to dissipate excess light energy that cannot be utilized in CO_2_ assimilation (Müller et al., 2001). Our results showed that under T_0_ condition, *F*_v_/*F*_m_ in water-deficient (W_−30_) plants significantly decreased by 16.7% compared with the normally-watered (W_0_) plants. There is no significant difference between the over-watered (W_+30_) plants and W_0_ plants. Moreover, there is a great change in *F*_m_ but not *F*_0_ under W_−30_ condition, suggesting that the decrease in *F*_v_/*F*_m_ was due to the decrease in *F*_m_. Except for *F*_v_/*F*_m_, there were no significant changes in the other chlorophyll fluorescence parameters such as *F*_v_′/*F*_m_′ and *q*_*P*_ in both W_−30_ and W_+30_ plants. The results indicate that (1) excess precipitation had no effect on *S. bungeana* at room temperature; (2) *S. bungeana* suffered from water deficit (decrease in *F*_v_/*F*_m_), and water stress inhibited plant's ability in thermal energy dissipation (Zivcak et al., 2014). This can be explained by the fact that an extreme decrease in trans-thylakoid pH gradient was not generated owing to cooperative consumption of light energy by CO_2_ fixation and photorespiration (Müller et al., 2001).

At the T_6_ condition, high temperature offset the negative effect of water deficit on *F*_v_/*F*_m_, and enhanced the positive effect of excess precipitation on *F*_v_/*F*_m_, *F*_v_′/*F*_m_′ and *q*_*P*_, leading to the increase in value. This indicates that the temperature higher by 6°*C* will be beneficial to the photosynthetic performance of *S. bungeana*. However, in the study by Xu and Zhou (2006), the combination of severe water stress and high temperature exhibited adverse effects on the PSII function of *Leymus chinensis*, which is similar to Petsas and Grammatikopoulos (2009)'s conclusion that PSII function of *Phlomis fruticosa* was progressively suppressed under long-term water deficit. This obvious difference may be explained by that *S. bungeana* can well adapt to stress environment for its well-developed root system (Cheng et al., 2012).

Furthermore, in the leaves of plants under optimum temperature and water condition, the mean value of *F*_v_/*F*_m_ was 0.678 (Table 1), which was lower than the typical value of 0.83 for non-photoinhibited leaves (Björkman and Demmig, 1987). There are two possible reasons to explain the difference. One is the usage of a different intensity of illumination during plants growing (Brestic et al., 2014) and the timing used for day/night regime was also different when compared to natural conditions. The other one is *S. bungeana* grown in the plastic pots with a small size under weak illumination may limit *S. bungeana*'s normal growth (Xu and Zhou, 2006). Therefore, the use of pots inside the greenhouse still requires further investigation.

**Table 1:** **Effects of precipitation treatments on maximum and minimum fluorescence yield in dark (*F*_m_ and *F*_0_, respectively), maximal quantum yield of PSII photochemistry for the dark-adapted state (*F*_v_/*F*_m_), coefficient of the photochemical quenching (*q*_*P*_) and maximal quantum yield of PSII photochemistry for the light-adapted state (*F*_v_′/*F*_m_′) of *Stipa bungeana* leaf under ambient temperature (T_0_) and high temperature (T_6_) conditions**.

**Temperature treatments**	**Water treatments**	**Chl fluorescence parameters**				
		***F*_m_**	***F*_0_**	***F*_v_/*F*_m_**	***F*_v_′/*F*_m_′**	***q*_P_**
T_0_	W_+30_	0.557 ± 0.130 ab	0.170 ± 0.048 a	0.691 ± 0.076 a	0.368 ± 0.109 a	0.627 ± 0.158 a
	W_0_	0.577 ± 0.121 a	0.181 ± 0.031 a	0.678 ± 0.078 a	0.426 ± 0.129 a	0.690 ± 0.150 a
	W_−30_	0.417 ± 0.181 b	0.172 ± 0.054 a	0.565 ± 0.096 b	0.448 ± 0.066 a	0.560 ± 0.195 a
T_6_	W_+30_	0.555 ± 0.090 a	0.164 ± 0.036 a	0.702 ± 0.058 a	0.524 ± 0.100 a	0.600 ± 0.088 a
	W_0_	0.548 ± 0.122 a	0.193 ± 0.069 a	0.652 ± 0.081 ab	0.461 ± 0.069 ab	0.461 ± 0.113 b
	W_−30_	0.522 ± 0.136 a	0.233 ± 0.112 a	0.569 ± 0.111 b	0.409 ± 0.129 b	0.400 ± 0.121 b

Different letters indicate significant differences (p < 0.05) between water treatments according Duncan test. Values shown are means ± standard deviation (SD) of nine to twelve replicates.

### Utilization of excess excitation energy under high temperature and abnormal water conditions

*F*_v_/*F*_m_ is known to be a sensitive indicator of plant photosynthetic performance (Björkman and Demmig, 1987). It reflects the maximum efficiency of photosynthetic apparatus converting the absorbed light energy into chemical energy, and has been widely used for the detection of photoinhibition (Dickmann et al., 1992; Herppich and Peckmann, 2000). The plants under stress have a lower value of *F*_v_/*F*_m_ than those under normal environment (Papageorgiou and Govindjee, 2004; Tuba et al., 2010; Shaw et al., 2014). Calatayud et al. (2013) proposed several reasons for why the *F*_v_/*F*_m_ ratio is preferable for the research of environmental stress. Firstly, it can be measured rapidly for the dark-adapted samples. Secondly, it is very useful for quick screening of stress-suffered plants in large quantities. Lastly, unlike the other parameters such as Φ_PSII_ or *q*_*P*_, it does not need an extended period of illumination as a single saturation pulse is enough. However, the decrease of *F*_v_/*F*_m_ can only reflect the degree of environmental stress, while the utilization of excess excitation energy is still unknown. To solve the problem, three fluorescence parameters which divides the allocation of absorbed light energy into three fractions: (1) utilized by PSII photochemistry (Φ_PSII_); (2) thermally dissipated via ΔpH and xanthophyll-dependent energy quenching (Φ_NPQ_); (3) non-regulated energy dissipation (Φ_NO_) (Demmig-Adams et al., 1996; Lazár, 2015). Among the latter three fluorescence parameters, Φ_PSII_ reflects light-induced protective mechanism while Φ_NO_ reflects a basal quenching which is not regulated by light. They shed a light on the study of plant's capacity to cope with excess excitation energy, and have been widely used to determine QA redox state and excitation energy fluxes in order to gain a better understanding of the stress response mechanisms (Calatayud et al., 2006; Osório et al., 2011).

**Figure 1:**
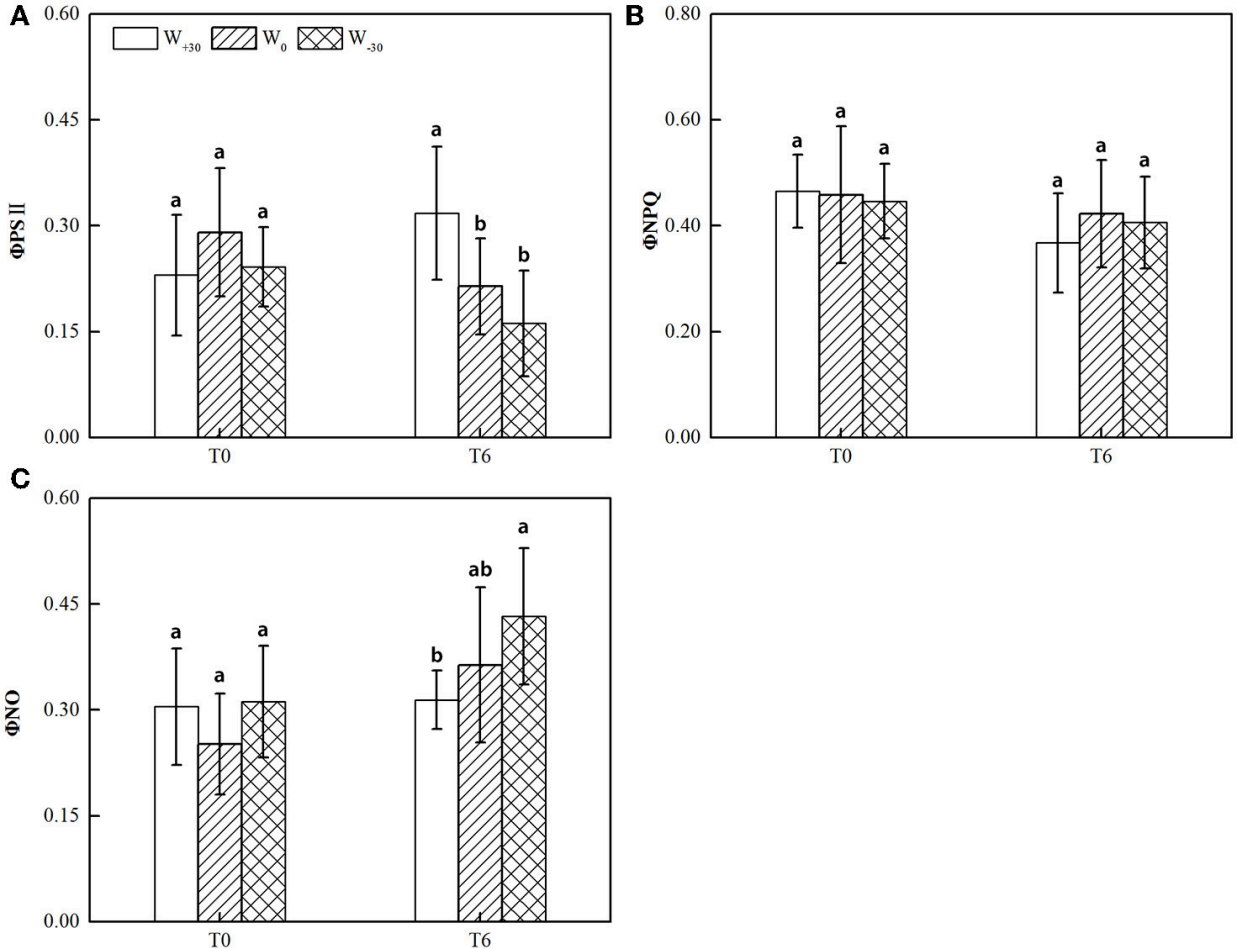
**(A)** Actual quantum yield of PSII photochemistry for the light-adapted state (Φ_PSII_), **(B)** quantum yield of regulated energy dissipation of PSII for the light-adapted state (Φ_NPQ_), **(C)** quantum yield of non-regulated energy dissipation of PSII for the light-adapted state (Φ_NO_) in the leaves of *S. bungeana* under two temperature treatments and three water treatments. Different letters indicate significant difference (*p* < 0.05) between water treatments in Duncan test. The values are expressed as mean ± standard deviation (SD), which was calculated with nine to twelve replicates.

As seen in Figure 1, the precipitation change had no significant effect (*p* > 0.05) on Φ_PSII_, Φ_NPQ_, and Φ_NO_ at T_0_ condition, though Φ_PSII_ decreased by 20.8 and 16.7% under W_+30_ and W_−30_ conditions and Φ_NO_ increased by 21.0 and 23.9%, respectively. At the T_6_ condition, high temperature slightly decreased the value of Φ_PSII_ at W_0_ and W_−30_ conditions. This suggests that heat dissipation of the excess light energy was activated to protect the photosynthetic apparatus from photoinhibitory damage (Ort and Baker, 2002; Omasa and Takayama, 2003). Whereas excess precipitation under T_6_ condition significantly increased Φ_PSII_, indicating that the increased precipitation can enhance the protective mechanism of PSII. In addition, Φ_NPQ_ showed no obvious change under all environment conditions and the change of Φ_NO_ was opposite to Φ_PSII_. This means that there was much overlap between Φ_PSII_ and Φ_NO_, indicating that energy dissipation by non-regulated quenching mechanisms tends to dominate the yield of PSII photochemistry under drought and heat stress, with the xanthophyll cycle-mediated thermal dissipation playing possibly a less important role. Similar results were also reported by Osório et al. (2011).

### Spatial heterogeneity of chlorophyll fluorescence parameters under various temperature and water conditions

**Figure 2:**
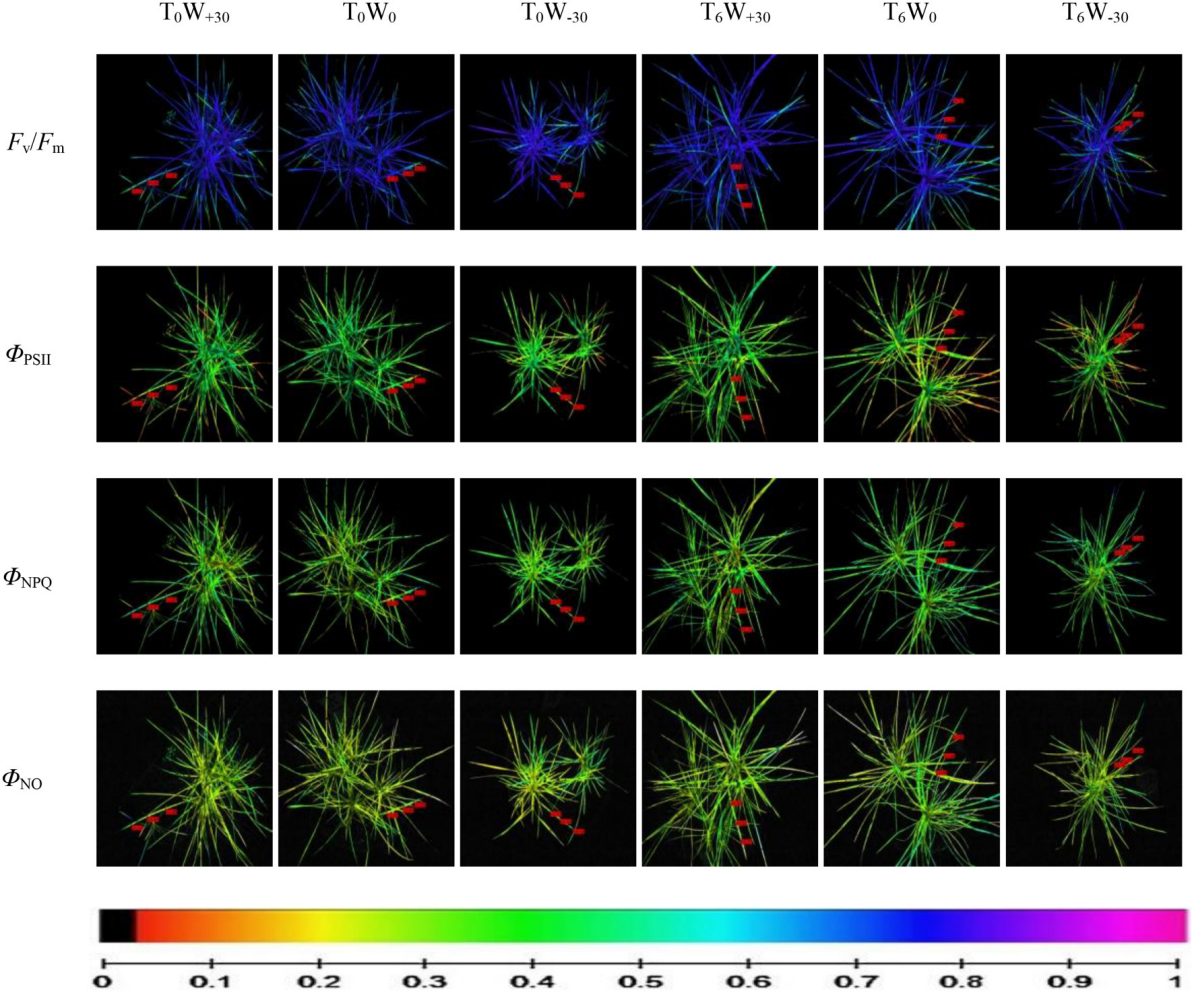
**Use of chlorophyll fluorescence imaging of whole plant *Stipa bungeana* under different temperature and precipitation conditions**. This figure illustrates several images of the same leaf of *Stipa bungeana* showing the spatial variation in the parameters Φ_PSII_, Φ_NPQ_, and Φ_NO_ at steady state with actinic illumination of 336 μmol photons m^−2^·s^−1^, and in the parameter *F*_v_/*F*_m_ after dark adaptation. The color scale showed at the bottom of the figure stands for values from 0 (black) to 1 (pink) based on Imaging Win v2.32 software. The three little red boxes in each image display the mean values of the selected fluorescence parameters within the AOI of one leaf.

In Table 2, the value of Chl a fluorescence was obtained from three leaves in each treatment. CFI reveals spatial changes in three areas of interest (AOIs) of the same leaves of *S. bungeana*, i.e., the bottom position of leaf, middle position of leaf and upper position of leaf as shown in Figure 2. For each AOI, the values of fluorescence parameter of all pixels within this area were averaged. In Figure 2, the images of a single leaf are used to show the heterogeneous distribution of light utilization (changes in Φ_PSII_, Φ_NPQ_, and Φ_NO_) and photosynthetic activity (change in *F*_v_/*F*_m_) over the surface of the whole leaf. The observation of changed image color is an intuitive process. Pixel-value images of *F*_v_/*F*_m_, Φ_PSII_, Φ_NPQ_, and Φ_NO_ were displayed with the help of a false color code, ranging from black (0.000) to pink (ending 1.000).

**Table 2:** **Effect of high temperature and precipitation change on maximal quantum yield of PSII photochemistry for the dark-adapted state (*F*_v_/*F*_m_), actual quantum yield of PSII photochemistry for the light-adapted state (Φ_PSII_), quantum yield of regulated energy dissipation of PSII for the light-adapted state (Φ_NPQ_), and quantum yield of non-regulated energy dissipation of PSII for the light-adapted state (Φ_NO_) of *Stipa bungeana* in different AOIs**.

	**Precipitation change**	**T**_**0**_				**T**_**6**_			
		***F*_v_/*F*_m_**	**Φ_PSII_**	**Φ_NPQ_**	**Φ_NO_**	***F*_v_/*F*_m_**	**Φ_PSII_**	**Φ_NPQ_**	**Φ_NO_**
AOI1	W_+30_	0.73 ± 0.02 a	0.31 ± 0.05 a	0.44 ± 0.04 a	0.25 ± 0.01 a	0.72 ± 0.05 a	0.37 ± 0.12 a	0.32 ± 0.07 a	0.31 ± 0.06 ab
	W_0_	0.76 ± 0.00 a	0.35 ± 0.05 a	0.42 ± 0.14 a	0.23 ± 0.08 a	0.71 ± 0.02 a	0.28 ± 0.06 a	0.44 ± 0.10 a	0.28 ± 0.03 b
	W_−30_	0.66 ± 0.02 b	0.30 ± 0.02 a	0.46 ± 0.06 a	0.24 ± 0.06 a	0.64 ± 0.10 a	0.23 ± 0.10 a	0.39 ± 0.09 a	0.38 ± 0.05 a
AOI2	W_+30_	0.54 ± 0.10 b	0.24 ± 0.05 ab	0.45 ± 0.08 a	0.31 ± 0.10 a	0.70 ± 0.04 a	0.31 ± 0.03 a	0.36 ± 0.06 a	0.33 ± 0.04 a
	W_0_	0.68 ± 0.02 a	0.31 ± 0.04 a	0.42 ± 0.11 a	0.27 ± 0.08 a	0.63 ± 0.11 a	0.19 ± 0.05 b	0.45 ± 0.02 a	0.36 ± 0.05 a
	W_−30_	0.73 ± 0.02 a	0.22 ± 0.03 b	0.47 ± 0.08 a	0.31 ± 0.05 a	0.53 ± 0.11 a	0.13 ± 0.03 b	0.43 ± 0.11 a	0.44 ± 0.12 a
AOI3	W_+30_	0.49 ± 0.04 a	0.14 ± 0.04 a	0.51 ± 0.08 a	0.35 ± 0.09 a	0.68 ± 0.09 a	0.27 ± 0.11 a	0.42 ± 0.13 a	0.31 ± 0.04 a
	W_0_	0.59 ± 0.03 a	0.21 ± 0.11 a	0.54 ± 0.15 a	0.26 ± 0.08 a	0.62 ± 0.09 a	0.17 ± 0.05 a	0.37 ± 0.16 a	0.46 ± 0.15 a
	W_−30_	0.61 ± 0.09 a	0.20 ± 0.06 a	0.41 ± 0.09 a	0.39 ± 0.04 a	0.53 ± 0.12 a	0.13 ± 0.03 a	0.40 ± 0.10 a	0.48 ± 0.12 a

Different letters indicate significant differences between water treatments at the same part of leaf (p < 0.05) according Duncan test. Values shown are means ± standard deviation (SD) of three replicates.

According to Table 2, it was found that CFI exhibited the spatial changes in different AOIs of the leaf of *S. bungeana*. Three different AOI were considered for each leaf. Each datum in the table is the mean value of the corresponding AOI from all leaves. At T_0_ condition, excess precipitation did not alter the *F*_v_/*F*_m_ in AOI1, but reduced the *F*_v_/*F*_m_ by 13.0 and 9.2% in AOI2 and AOI3, respectively. Water deficit significantly decreased the *F*_v_/*F*_m_ by 15.0% in AOI1, but there was no significant change in AOI2 and AOI3. At T_6_ condition, excess precipitation increased the *F*_v_/*F*_m_ in AOI1, AOI2, and AOI3 by 2.8, 12.6, and 9.0%, respectively. This means that a 6°*C* higher temperature is beneficial for *F*_v_/*F*_m_ under abundant water condition. In contrast, water deficit decreased the *F*_v_/*F*_m_ in AOI1, AOI2, and AOI3 by 9.2, 14.9, and 16.8%, respectively. This suggests a reduction in light energy utilization by chloroplasts in the photosynthesis. According to the changes of *F*_v_/*F*_m_ and the results of One-way ANOVA in Table 2, it can be concluded as follows. The middle position of leaf (AOI2) is most sensitive to excess precipitation under both T_0_ and T_6_ condition, while the bottom position (AOI1) and upper position (AOI3) are most sensitive to water deficit under both temperature conditions.

The contribution of different pathways to energy partitioning in PSII complexes is shown in Table 2. In AOI1 and AOI3, the actual quantum yield of PSII photochemistry for the light-adapted state (Φ_PSII_) which can indirectly reflect linear electron transport was not affected by precipitation change at both T_0_ and T_6_ treatment. This confirms that photoinhibition is not induced under these conditions. In AOI2, high temperature (T_6_) improved the effect of W_+30_ on *F*_v_/*F*_m_ by 63.6%. This change of Φ_PSII_ resulted from changes in the total non-photochemical quenching capacity (Φ_NPQ_+ Φ_NO_). The quantum yield of regulated energy dissipation (Φ_NPQ_) was quite similar in all AOIs under different environment conditions, indicating that no excess light energy was produced by precipitation change and high temperature. The values of quantum yield of non-regulated energy dissipation (Φ_NO_) were low and similar in all AOIs at T_0_. This means that there were sufficient photochemical conversion and protective regulatory mechanisms in the whole leaf. At T_6_ condition, water deficit increased Φ_NO_ in whole leaf, indicating that high temperature exacerbated the negative effect of water deficit on energy dissipation. Both photochemical energy conversion and protective regulatory mechanism were not enough.

The Two-way ANOVA (Table 3) indicated that, *F*_v_/*F*_m_ was significantly influenced by precipitation change at T_0_ condition and varied greatly at different AOIs (*p* < 0.01), exhibiting significant interaction of AOI and precipitation change (*p* < 0.01). The other chlorophyll fluorescence parameters such as Φ_PSII_ and Φ_NO_ exhibited significant difference across AOIs, but were not affected by precipitation change. Under condition, only Φ_PSII_ was significantly affected by both AOI and precipitation change, but the interaction was not significant (*p*>0.05). To conclude, *F*_v_/*F*_m_ is most sensitive to precipitation change at T_0_ condition, while Φ_PSII_ is the most sensitive indicator at T_6_ condition. However, in the study by Lazár et al. (2006), even if there are no changes in the mean value of a fluorescence parameter, there can be the changes in shapes of statistical distributions of fluorescence parameter which is an early indication of a plant stress. Base on this, we should not only find the most sensitive parameter (Kalaji et al., 2014) and the most sensitive species (Swoczyna et al., 2010) but also find the best (statistical) method for detection of the stresses is more important. The use of fluorescence imaging and the detection of photosynthetic performance of *Stipa bungeana* response to climatic change still requires further investigation.

**Table 3:** **Multiple range test among effects of areas of interest (AOI) and watering treatments on *Stipa bungeana* leaf Chl fluorescence parameters under ambient temperature (T_0_) and high temperature (T_6_) conditions based on the Two-way ANOVA**.

		***F*_v_**/***F*_m_**			**Φ**_**PSII**_			**Φ**_**NPQ**_			**Φ**_**NO**_		
		***df***	***F***	***P***	***df***	***F***	***P***	***df***	***F***	***P***	***df***	***F***	***P***
T_0_	AOI	2	20.918	0.000	2	14.455	0.000	2	0.587	0.566	2	3.815	0.042
	Precipitation	2	8.041	0.003	2	3.051	0.072	2	0.090	0.914	2	1.879	0.182
	AOI × Precipitation	4	5.160	0.006	4	0.692	0.607	4	0.847	0.514	4	0.648	0.635
T_6_	AOI	2	2.166	0.144	2	4.713	0.023	2	0.223	0.802	2	2.661	0.097
	Precipitation	2	5.272	0.016	2	10.366	0.001	2	0.717	0.502	2	4.622	0.024
	AOI × Precipitation	4	0.198	0.936	4	0.075	0.989	4	0.661	0.627	4	0.926	0.471

df, Degree of freedom; F, F-value, used for Homogeneity of variance test; P, Significant level.

## Conclusion

Chlorophyll fluorescence imaging provided detailed intuitive information on the spatial heterogeneity of chlorophyll fluorescence parameters of *S. bungeana* and facilitated the investigation of plant photosynthetic performance under various temperature and water conditions. Our results showed that *S. bungeana* has strong ability in protecting photosynthetic apparatus against the photoinhibitory damage from drought, and a 6°*C* higher temperature could offset the negative effect of water deficit to a certain extent. On the other hand, excess precipitation had no significant effect on PSII at room temperature. But high temperature had a positive effect on PSII and significantly enhanced the photosynthesis of *S. bungeana*. We also found that it is energy dissipation by non-regulated quenching mechanisms rather than the xanthophyll cycle-mediated thermal dissipation that plays an important role in dominating the yield of PSII photochemistry under climate change. This study also found that *F*_v_/*F*_m_ measured in AOIs was the most sensitive indicator to precipitation change under room temperature, while Φ_PSII_ is more sensitive to precipitation change at higher temperature.

## Author contributions

GZ and YW conceived the experiment, YW, XL, and ZX conducted the experiment, XS analyzed the results and wrote the manuscript. All authors reviewed the manuscript. No conflict of interest exits in the submission of this manuscript, and the manuscript is approved by all authors for publication. We would like to declare that the work described was original research that has not been published previously, and not under consideration for publication elsewhere, in whole or in part.

## Funding

This work was supported by the national Basic research Program of China [grant number 2010CB951301] and Chinese Academy of Sciences “Strategic Priority Research Program-Climate Change: Carbon Budget and Relevant Issues” [grant number XDA-05050408].

### Conflict of interest statement

The authors declare that the research was conducted in the absence of any commercial or financial relationships that could be construed as a potential conflict of interest.

## Abbreviations

AOI: areas of interest
Chl: chlorophyll
CFI: chlorophyll fluorescence imaging
PSII: photosystem II
*F*_v_/*F*_m_: maximal quantum yield of PSII photochemistry for the dark-adapted state
qP: coefficient of the photochemical quenching
Φ_PSII_: actual quantum yield of PSII photochemistry for the light-adapted state
Φ_NPQ_: quantum yield of regulated energy dissipation of PSII for the light-adapted state
Φ_NO_: quantum yield of non-regulated energy dissipation of PSII for the light-adapted state
*F*_v_′/*F*_m_′: maximal quantum yield of PSII photochemistry for the light-adapted state.
